# The complete chloroplast genome sequence of *Rotheca myricoides* (Hochst.) Steane & Mabb., a traditional medicinal plant

**DOI:** 10.1080/23802359.2021.1966340

**Published:** 2021-08-18

**Authors:** Xiaoai Fang, Siyi Zhang

**Affiliations:** Xi’an International University, Xi’an, China

**Keywords:** *Rotheca myricoides* (Hochst.) Steane&Mabb., chloroplast genome, illumina sequencing, phylogenetic analysis

## Abstract

*Rotheca myricoides* (Hochst.) Steane & Mabb. is a plant species used in traditional medicine for the management of diabetes in the lower eastern part of Kenya (Kitui, Machakos and Makueni Counties, Kenya) that is mainly inhabited by the Kamba community. The complete chloroplast genome sequence of *R. myricoides* was assembled from the whole genome Illumina sequencing data. The genome was 150,596 bp in length, contained an SSC region of 17,237 bp and LSC region of 83,489 bp, separated by IRs of 24,935 bp, each. The genome contained 114 unique genes, including 80 PCGs, 4 rRNA genes, and 30 tRNA genes. In addition, 18 genes contained one or two introns, including 10 PCG genes with a single intron, 2 PCG genes harboring two introns, and 6 tRNA genes harboring a single intron. Phylogenetic analysis supported *R. myricoides* had the closest genetic relationship with *Rotheca serrata* and clustered with the Rotheca family species.

*R. myricoides* is used globally as a traditional medicinal plant species belonging to the genus *Rotheca* the largest genus of the family Labiatae (Bashwira and Hootele 1988). It is used for management of diabetes in the lower eastern part of Kenya. A leaf decoction is prepared by boiling and a cup (250 ml) is taken daily (Keter and Mutiso 2012). Other ethnobotanical uses of this species include epilepsy, arthritis, typhoid, cough, eye problems, tonsillitis, rheumatism, gonorrhea (Moshi et al. 2012), cancer (Esubalew et al. 2017), malaria (Mukungu et al. 2016), dysmenorrhea, sterility, impotence, cough, furunculosis, inflammation, and snakebites (Richard et al. 2011). It is traditionally brewed and drank as a tea product in Asian countries to relieve swelling and pain. In China, *R. myricoides* is mainly distributed in Guangdong provience. In this study, the complete plastid genome sequence of *R. myricoides* is a useful resource and provides additional valuable data for the phylogenetic study of Rotheca in the future.

The fresh leaves of *R. myricoides* were collected in Zhanjiang (110.3 E, 21.2 N; Guangdong, China), and the voucher specimen (RM772543) deposited at Pharmaceutical Laboratory in Xi'an International University (Siyi Zhang, 906642120@qq.com). The total genomic DNA was extracted with modified CTAB protocol. Subsequently, the integrity and quality of the DNA werechecked through agarose gel electrophoresis and spectrophotometry using a Nanodrop 2000 instru-ment (Thermo Scientific, DE, USA), respectively. Then the genomic DNA of *R. myricoides* was used for the subsequent shotgun library construction and High-throughput sequencing on the Illumina HiSeq X Ten Sequencing System (Illumina, CA, USA).

The data were assembled *R. myricoides* chloroplast genome sequence with length 150, 596 bp. The cp genome was showed a typical quadripartite structure, consisted of a large single copy region with 83,489 bp (LSC) and small single copy region with 17,237 bp (SSC), and separated by a pair of inverted repeatregions with 24,935 bp (IRs), each. The whole genome sequence GC content is 38.6%, while in LSC, SSC and IRs (IRA + IRB) are 36.37%, 32.8% and 43.7%, respectively. A total of 113 genes were predicted in the whole chloroplast genome of *R. myricoides,* including 80 protein-coding genes, 29 tRNA genes, and 4 rRNA genes. In addition, 18 genes contain one or two introns, which include 10 PCG genes (*atp*F, *ndh*A, *ndh*B, *pet*B, *pet*D, *rpl*2, *rpl*16, *rpo*C1, *rps*12, *rps*16) possessing a single intron, 2 PCG genes (*clp*P, *ycf*3) harboring two introns, and 6 tRNA genes (*trn*A-UGC, *trn*G-UCC, *trn*I-GAU, *trn*K-UUU, *trn*L-UAA, *trn*V-UAC) harboring a single intron.

To ascertain its phylogenetic placement within the family Rotheca, the Bayesian Inference (BI) phylogenetic tree was reconstructed with the MRBayes V3.1.2 (Ronquist and Huelsenbeck 2003) integrated with Topali V2.5 (Milne et al. 2009) based on 15 genes (*atp*A, *atp*B, *mat*K, *ndh*A, *ndh*H, *psa*B, *psb*A, *psb*B, *psb*C, *psb*D, *rbc*L, *rpo*A, *rpo*B, *rpo*C1 & *rpo*C2) in the complete chloroplast genome of *R. myricoides* and 26 other species. As expected, *R. myricoide* was close to *R. serrata* formed a strongly monophyletic clade (Figure 1). Our findings suggest that the high-level taxonomy of the family Rotheca may need further investigations.

**Figure 1.**
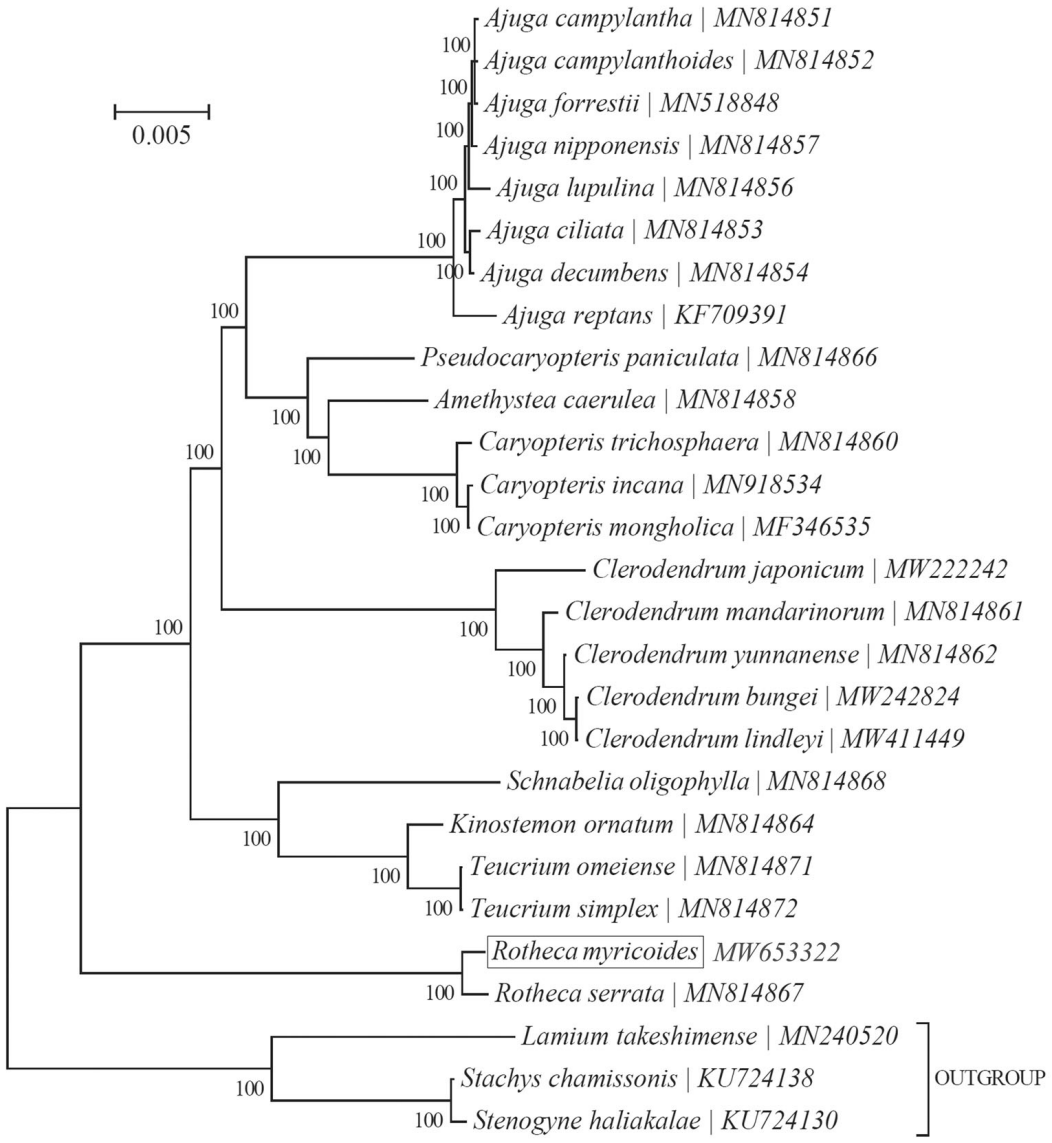
Bayesian inference (BI) phylogenetic analysis based on 27 complete chloroplast genomes. The bootstrap values were based on 1000 resamplings and are placed next to the branches.

## Data Availability

The data that support the findings of this study are openly available in the US National Center for Biotechnology Information (NCBI database) at https://www.ncbi.nlm.nih.gov/, reference number: MW653322, and the associated BioProject, SRA, and Bio-Sample numbers are PRJNA724817, SRR14322715 and SAMN18858889, respectively.
